# Neural networks and hospital length of stay: an application to support healthcare management with national benchmarks and thresholds

**DOI:** 10.1186/s12962-021-00322-3

**Published:** 2021-10-09

**Authors:** Roberto Ippoliti, Greta Falavigna, Cristian Zanelli, Roberta Bellini, Gianmauro Numico

**Affiliations:** 1 Faculty of Business Administration and Economics, Bielefeld University, Bielefeld, North Rhine-Westphalia Germany; 2 Research Institute on Sustainable Economic Growth (IRCrES), National Research Council of Italy (CNR), Moncalieri, TO Italy; 3 Quality and Management Control Unit, Azienda Ospedaliera SS Antonio e Biagio e Cesare Arrigo, Alessandria, AL Italy; 4 Medical Oncology Unit, Azienda Ospedaliera Santa Croce e Carle, Cuneo, CN Italy

**Keywords:** Neural Networks, Hospital admission, Length of stay, Health services research

## Abstract

**Background:**

The problem of correct inpatient scheduling is extremely significant for healthcare management. Extended length of stay can have negative effects on the supply of healthcare treatments, reducing patient accessibility and creating missed opportunities to increase hospital revenues by means of other treatments and additional hospitalizations.

**Methods:**

Adopting available national reference values and focusing on a Department of Internal and Emergency Medicine located in the North-West of Italy, this work assesses prediction models of hospitalizations with length of stay longer than the selected benchmarks and thresholds. The prediction models investigated in this case study are based on Artificial Neural Networks and examine risk factors for prolonged hospitalizations in 2018. With respect current alternative approaches (e.g., logistic models), Artificial Neural Networks give the opportunity to identify whether the model will maximize specificity or sensitivity.

**Results:**

Our sample includes administrative data extracted from the hospital database, collecting information on more than 16,000 hospitalizations between January 2018 and December 2019. Considering the overall department in 2018, 40% of the hospitalizations lasted more than the national average, and almost 3.74% were outliers (i.e., they lasted more than the threshold). According to our results, the adoption of the prediction models in 2019 could reduce the average length of stay by up to 2 days, guaranteeing more than 2000 additional hospitalizations in a year.

**Conclusions:**

The proposed models might represent an effective tool for administrators and medical professionals to predict the outcome of hospital admission and design interventions to improve hospital efficiency and effectiveness.

## Background

Hospital admission is a costly and limited resource [1], but it is also a necessary step in the trajectory of most diseases. As outpatient care and home care have been financed and developed, inpatient care has evolved into a high-technology, high-intensity, multidisciplinary intervention. Hospital networks are adapting to this evolution, with progressive concentration of skills and structures and unavoidable transformations of their internal organization [2]. Although clinicians are often inclined to resist this change, they should see it as an opportunity for improvement and professional enhancement. Optimal clinical care should be pursued together with efficiency and frugality in the use of resources. In fact, these two purposes are not contradictory, since shorter hospital stays have obvious economic effects but, when associated with a rigorous clinical pathway, they also have huge health benefits, e.g., limiting the risks of hospital infection [3], thrombosis [4], and reduced physical autonomy [5]. Moreover, inefficiency invariably causes reduced accessibility: overcrowding of emergency departments and medical units is an alarming cause of delayed access to life saving services. Smooth in-hospital flows require a complex mix of conditions that have to come together in the pursuit of a shared goal. These conditions include: clinical choices, logistics, information technology and quality of health records, connections between units and services, and also personal motivation and team working. At the basis of any effort to change, however, there is the ability to select measurable indicators, together with tools for predicting the type of hospital stay and its possible outcomes, and this is precisely the purpose of our work.

### Literature review: length of stay and prediction models

Length of stay (LOS) is one of the most widely used and easily available efficiency indicators [6]. This indicator assesses the speed of the clinical pathway and has the advantage of being uniformly measured and quite comparable. In the last two decades, several prediction models have been developed by scholars using consolidated techniques (e.g., Markov models, Multistage models, Discrete-Event models) to study LOS and the impact of selected covariates on this outcome (e.g., sex, age, admission method, diagnosis, severity of illness, hospital characteristics). As for the observations under investigation, the literature is quite heterogeneous, focusing on specific medical units or departments, as well as single hospitals or the whole healthcare system. Some authors adopt a continuous-time hidden Markov model with discrete states to model inpatient behavior, representing the flow of patients around departments of geriatric medicine and investigating the effect of two selected covariates on occupancy time, i.e., age and sex [7]. Other authors propose a Gamma mixture risk-adjusted model to analyze maternity LOS within obstetrical Diagnosis Related Groups (DRGs) and connected determinants, i.e., patients’ demographic characteristics, health provision factors, and other clinical and hospital factors [8]. In both cases, the determination of pertinent factors would help hospital administrators and clinicians to efficiently manage LOS and expenditure. Taking a Texas Medical Center in Houston into account, Kapadia and colleagues model the flow of patients in a pediatric intensive care unit using a discrete Markovian process [9]. The aim of their work is to represent the flow of patients in relation to their illness (measured through the Pediatric RISk of Mortality, PRISM) in order to minimize total costs associated with hospitalization. Note that the PRISM score is based on seven physiological and seven laboratory variables, each reflecting an organ system dysfunction, which are combined together into a score that represents a proxy for the severity of the illness and the risk of mortality in the current hospitalization [10]. The results suggest that stochastic models, like Markov chains and Monte-Carlo simulations, can be very successful in assessing LOS if the illness risk is well defined.

Jeon and colleagues propose another study, with practical values that are useful in resource planning, relying on 5 years’ retrospective data for patients admitted to the Belfast City Hospital with a diagnosis of stroke [11]. Using a phase-type recovery model, they investigate LOS according to patients’ age, type of stroke (i.e., hemorrhagic, cerebral infarction, and transient ischemic attack) and mode of discharge (i.e., the patient may die, be transferred to a nursing home, or be discharged to the individual’s usual residence). On the other hand, Akkerman and Knip analyze the department of cardiac surgery of a Dutch hospital, looking at the relationships among patients’ LOS, beds availability, and hospital waiting lists [12]. In detail, they investigate patients’ LOS in hospital wards following cardiac surgery, describing and evaluating several scenarios for hospital management using Markov chain theory and simulation experiments.

Taking a large integrated healthcare delivery system into account (more than 300,000 hospitalizations occurring over 2 years in 17 hospitals), Harrison and Escobar adopt Multistage models to describe LOS distributions, trying to establish whether such models could be used for patient groups restricted by diagnosis, severity of illness, and the hospital supplying the treatments [13]. Considering the Irish healthcare system and focusing on delayed discharges, Rashwan and colleagues illustrate a system dynamics methodology used to model the flow of elderly patients, in order to better understand the dynamic complexity behind this phenomenon [14]. According to their results, there is evidence that the proposed system dynamic methodologies can support healthcare management in analyzing both different care pathways and delayed discharges of patients, optimizing their LOS. Finally, concentrating on elderly patients treated by the Regional Healthcare System of Italy’s Abruzzo Region, Gordon and colleagues introduce a new methodology, based on a series of conditional Coxian phase-type distributions, that clusters patients according to their covariates (i.e., age, gender, and admission method) and LOS in hospital and then models patient pathways, making it possible to predict their LOS in hospital and community care [15]. This approach can shed new light on the rates at which patients move between healthcare suppliers (i.e., hospital and community care), supporting managers in reducing the negative effects of bed occupancy and of the premature discharge of patients without a suitable period of convalescence.

Clearly, according to the aforementioned literature, the problem of correct inpatient scheduling is relevant for healthcare management. On the one hand, the care pathways and internal organization of beds can affect the patients’ chances to receive treatment and be hospitalized. On the other hand, bed occupancy represents a missed opportunity to increase hospital revenues by means of other treatments and/or hospitalizations, increasing the supply of healthcare services on the market. Considering public healthcare systems, this means longer waiting lists and growing inefficiency, which policy makers will have to face. Although the literature proposes interesting and effective methods to identify the best settings and approaches to managing beds [16, 17], there is a gap concerning the latter point, i.e., estimating the opportunity cost of such interventions, and this is a fundamental step to support the implementation of the proposed interventions by policy makers. With the purpose of filling this knowledge gap, our paper applies a deep learning technique, i.e., Artificial Neural Networks (ANNs) to identify inpatients with a negative outcome and then, assuming effective interventions by the medical professionals, quantifies the increase in revenues for the additional hospitalizations that might be achieved. According to Shahid and colleagues, ANNs are leveraging machine-learning techniques that can support physicians with their diagnosis, as well as managers to support their decisions and to improve delivery of efficient care [18]. Indeed, according to Shahid and colleagues, ANNs-based solutions applied on the meso- and macro-level of decision-making suggests the promise of its use in contexts involving complex, unstructured or limited information, supporting an effective and efficient supply of care by medical institutions.

In detail, our work analyzes LOS according to selected covariates extracted from an administrative hospital database, adopting national benchmarks and thresholds to identify “too long” hospitalizations and presenting predictive models based on ANNs. Both the methodology applied to the case study (i.e., ANNs) and the dataset used (with more than 16,000 hospitalizations and 20 covariates) represent further contributions to the current knowledge, supporting both hospital administrators and clinicians in efficiently managing LOS and expenditure. Indeed, these prediction models may represent an effective tool for administrators and medical professionals to predict the outcome of hospital admission, selecting the patient groups at higher risk of negative outcomes, and to design interventions to improve hospital efficiency.

## Methods

We investigate the Department of Internal and Emergency Medicine (DIEM) of a general hospital located in the North-West of Italy. In detail, the DIEM is structured around 13 clinical units: cardiology, hematology, geriatrics, infectious diseases, internal medicine, emergency medicine, nephrology, neurology, coronary care, gastroenterology, oncology, respiratory diseases, and rheumatology.

Administrative data were extracted from the hospital database, collecting information on more than 16,000 hospitalizations between January 2018 and December 2019. Then, the data were combined together (as inputs or outputs), applying ANNs, to create prediction models that may be adopted by physicians to support their managerial and clinical decision making. Such decision-making support, based on ANNs, may be able to identify patients with negative outcomes, guiding physicians and managers in the necessary interventions. In detail, considering hospitalizations in 2018, we performed a macro analysis on the whole sample without any distinctions among clinical units (i.e., the whole DIEM) and a micro analysis on the main clinical units and their hospitalizations. Lastly, we took hospitalizations in 2019 into consideration and tested the proposed prediction models, estimating expected clinical and managerial benefits.

### Artificial Neural Networks (ANNs)

ANNs are complex models organized in layers, (multilayer) formed by neurons (also called perceptrons) and interconnected via synapses (weights). Due to their well-known ability to generalize behaviors [19], ANNs have been successfully applied to many clinical fields [18], such as urinary tract infections and celiac disease [19, 20], total hip replacement surgery [21], early ruling-in/ruling-out of patients with suspected acute myocardial infarction using frequent biochemical monitoring [22], as well as in hospital management such as, for example, to evaluate patients’ admissions to EDs [23, 24], patients’ readmission [25], and patients’ appointment scheduling [26].

**Fig. 1:**
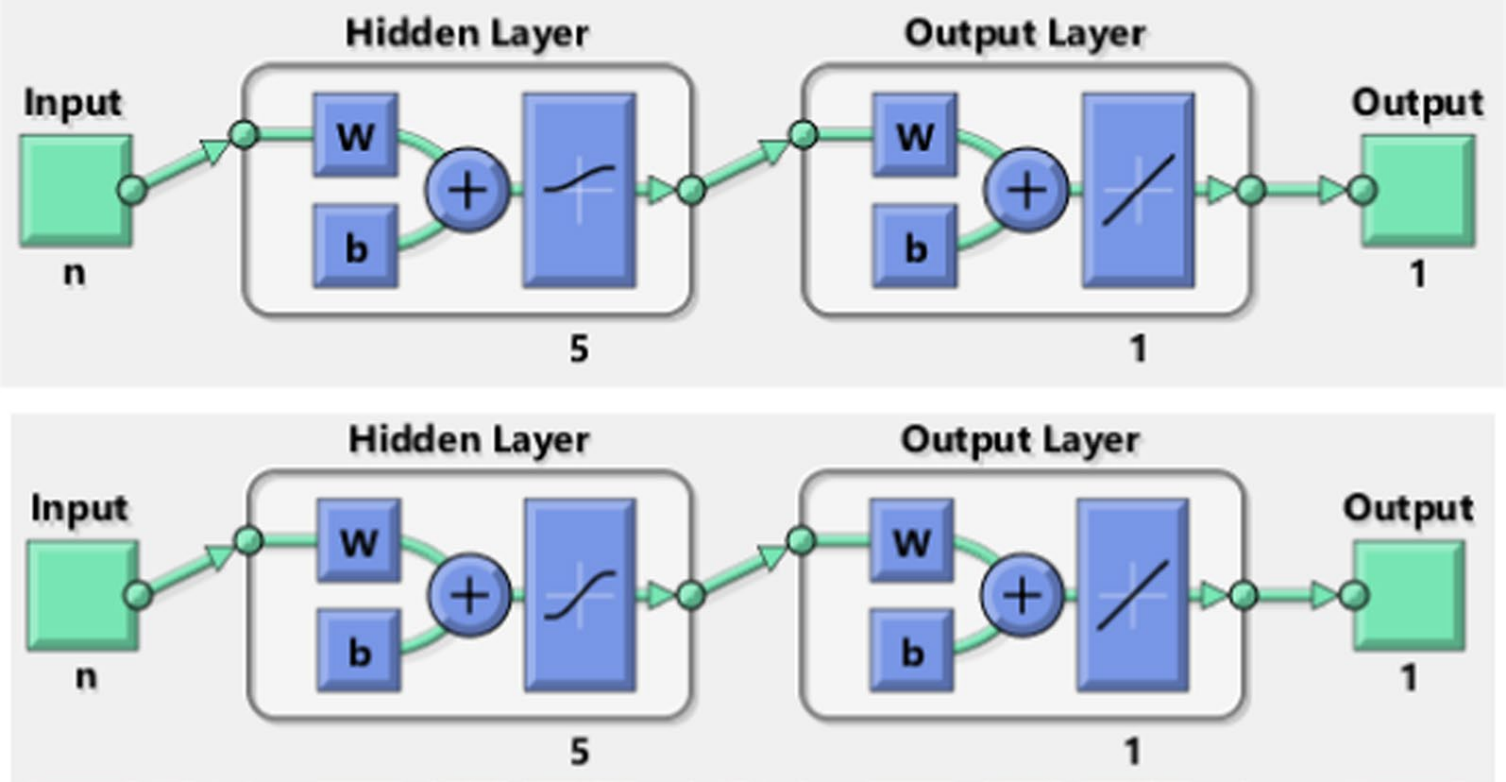
Artificial Neural Network architectures (*logsig and tansig functions*)

As highlighted in Fig. 1, the first layer is called “input layer” and it is composed of a number of neurons (or nodes) equal to that of the variables analyzed (in our specific case, as many neurons as the available pieces of information on the patients). The last layer is the “output layer”, from which the results of the models are derived. The number of nodes in this layer depends on the type of answer expected. Typically, there is only one neuron because the result is expressed in dichotomous form. Between the input layer and the output layer there are hidden layers, which can be more than one. The literature points out that a single hidden layer can approximate any functional form [27, 28]. The number of neurons in the hidden layers must be found empirically [29, 30], although some authors have tried to define specific rules [31–33].^1^ The links between the layers are the “synapses”, mathematically called weights, which collect information about the relationships between the input variables and the expected outputs. These relationships are formalized through activation functions that are generally non-linear in the first layer (i.e., tansig, logsig, hardlim, and so on). In our model, these functions are non-linear from the input layer to the hidden one (tansigmoidal in the first architecture and logsigmoidal in the second one)^2^ and linear from the hidden layer to the output one. The relationships between the layers are collected in weight matrixes and their analysis makes it possible to evaluate the contribution of each piece of information to the definition of the expected outputs [34–36]. The MultiLayer Perceptron (MLP) network is represented in Fig. 1 and its links are feed-forward because the connections come from the input layer to the hidden one and from the hidden layer to the output one. Backward or recursive relationships are not considered in this framework. The feed-forward MultiLayer Perceptron works with a supervised learning technique through a back-propagation algorithm. Note that there are some network frameworks that use an unsupervised procedure, i.e., Self-Organizing Map or Kohonen networks [38].

The supervised learning with the back-propagation algorithm works following this procedure: the initial sample is divided into two sub-samples, i.e., the training sample and the validation sample. In the first phase, only elements from the training sample are introduced into the model and, through the back-propagation algorithm, the network attempts to minimize the mean-square output error over the entire training set. The ANN computes weights matrixes until a predefined error threshold is reached [37]. In this step, the model is fed information about the patients but also about the selected target. This stage is very important because the ANN learns from the data and collects, within weight matrixes, information about the relationships between the variables. It clearly emerges that dividing the initial sample into training and validation is critical: the training set must represent all possible types of patients with their specific characteristics. In our case, we adopt the following proportions: 2/3 in the training sample and 1/3 in the validation sample.

Once the weights and ANN framework are defined (i.e., type of activation functions between layers; number of hidden layers and their nodes; other technical parameters, such as the search function for the optimal gradient, etc.), these parameters are applied to the validation sample, which is introduced into the ANN without any information on the selected outcomes. The ANN applies the framework to the new data in order to evaluate results and the ability of the model to provide correct classification. In our model, information about the patients is introduced into the input layer with the aim to obtain an outcome for each patient, indicating whether the selected outcomes are likely (1 if they are, 0 if they are not).

Afterwards, we apply the algorithm aimed at maximizing either the specificity or the sensitivity value of our model, i.e., we can decide whether to maximize the network’s ability to identify the number of true negatives or true positives [38]. Concerning computational issues of the algorithm, a bootstrap procedure is used in this study in order to improve the robustness of estimates. Moreover, since in the training phase ANN weights are randomly set every time, that algorithm allows us to run the ANN for a defined number of times (i.e., 100 replications). Finally, the algorithm presented here runs in the training phase and yields network parameters trying to ensure, if possible, minimum levels of sensitivity (i.e., 0.80) and specificity (i.e., 0.70) defined at the beginning of the computation. Thus, when a physician introduces new patient data into the model to forecast a negative outcome, the answer obtained about a potential future event is specifically based on levels of sensitivity and specificity set ex-ante. Analyses were performed using the MATLAB (release R2019b, 64bit) software.

### Applications with smart solutions

The main application of this new technology is the adoption of smart solutions (e.g., a mobile app) to customize the stratification of patients admitted to the DIEM [39]. Clinical information about the patients might be collected at his/her admission to the Department and then processed by the algorithm based on ANNs to support the decision making process regarding hospitalization and specialist investigations. On the one hand, the adoption of these smart solutions gives the opportunity to customize risk stratification in real time, according to the specific clinical case (i.e., the patient’s health status). On the other hand, further collection of data about incoming patients makes it possible to gather new evidence to refine the algorithm, so that updated versions of our innovative technology will become ever more effective at every access. In other words, ANNs and its application to smart solutions can provide an effective learning decision-making system to support health services.

### Input and output specification

According to the empirical strategy and the proposed methodology based on ANNs, two model definitions have to be identified, i.e., which inputs and outputs are adopted for the macro and micro analysis respectively. In both analyses, two outcomes are introduced:

- long hospitalization, which is a dummy variable equal to 1 if the hospitalization is longer than the national average, 0 otherwise;
- outlier hospitalization which is a dummy variable equal to 1 if the hospitalization is longer than a specific reference value, 0 otherwise.

In the estimation of these outcomes, we consider all types of hospital discharges (e.g., to the patient’s home, to another hospital, as well as death). The former outcome is based on a dynamic and variable benchmark, which is equal to the average of all hospitalizations occurred under a specific DRG code for a selected year (data source: Annual Report published by the Italian Ministry of Health, 2018). The latter outcome is based on a static threshold, which has been fixed for every specific DRG by the Italian Ministry of Health in 2008 through a dedicated regulation (i.e., *Decreto Ministero della Salute del 18 Dicembre 2008*). Obviously, the threshold is higher than the benchmark, which means that the outliers are a sub-sample of the long hospitalizations, and this is exactly why we decided to adopt both outcomes in our analysis, so that we may be able to estimate an interval for our potential interventions (i.e., between the benchmark and the threshold).

For what concerns the macro analysis, that is to say, the model specification in which we consider the whole sample of observations without any distinctions regarding departments (i.e., among clinical units), the inputs are the following:

- sex, which is a dummy variable equal to 1 if the patient is male, 0 otherwise;
- age, a matrix of four dummy variables according to the seniority class of patients, i.e., “18–40”, “41–65”, “66–75”, “> 75”;
- presence of cancer, which is a dummy variable equal to 1 if the patient has received a diagnosis of cancer;
- type of admission, which is a matrix of three dummy variables according to the access, i.e., “urgent hospitalization with direct access”, “urgent hospitalization from the emergency rooms” or “planned hospitalization”;
- time slot, which is a matrix of three dummy variables according to the access, i.e., “morning”, “afternoon” or “night”;
- day of admission, which is a matrix of seven dummy variables according to the access, ranging from Monday to Sunday.
- principal discharge diagnosis, which is a matrix of eleven dummy variables according to general areas of these diagnosis, i.e., selecting the 10 classes with the highest number of cases, which represent at least over 80% of the observations, plus one residual dummy variable.

In the micro analysis, that is to say, the model specification in which we look at single clinical units and their hospitalizations, the inputs are the same as in the macro analysis, but the selected top 10 diagnosis classes, which are more specific in this case, and the presence of internal transfers among the same units are also investigated. Note that we use the clinical unit to which patients are first admitted as our selection criterion. Finally, note that the micro analysis focuses on the most representative clinical units, i.e., internal medicine, cardiology, emergency medicine, geriatrics, respiratory diseases, neurology, and oncology. Obviously, taking the 2018 hospitalizations into account, one model for each clinical unit is run in order to collect specific weights that can explain the relation between inputs and outcomes.

### Empirical strategy

The results of the prediction models computed according to the aforementioned specifications are evaluated on the basis of the following indexes:

- incorrect classification, i.e., the proportion of actual positives and actual negatives that are not correctly identified as such, in relation to the selected outcome and the whole population of patients;
- sensitivity, i.e., the proportion of actual positives that are correctly identified as such, in relation to the selected outcome and the population of positive patients;
- specificity, i.e., the proportion of actual negatives that are correctly identified as such, in relation to the selected outcome and the population of negative patients;
- false positive rate (Type I error), i.e., the proportion of patients identified as false positives, in relation to the selected outcome and the population of actual negative patients;
- false negative rate (Type II error), i.e., the proportion of patients identified as false negatives, in relation to the selected outcome and the population of actual positive patients;
- Positive Likelihood Ratio, i.e., the change in pre-test probability caused by a positive test results, with values that range between 1 and + ∞ (the higher, the better);
- Negative Likelihood Ratio, i.e., the change in pre-test probability caused by a negative test results, with values that range between 0 and 1 (the lower, the better);
- Area Under the Curve, i.e., the area under the receiver operating characteristic curve, with values that range between 0 and 1 (the higher, the better).

These indexes represent the expected quality of our prediction models, i.e., the ability of our models to correctly identify patients in relation to the selected outcome. Finally, to complete the analysis, we have also estimated the Garson index for each variable introduced into the ANN, which represents its percentage contribution to the outcomes under investigation [35], as well as the expected sign of their contribution according to the NN synaptic weights [40].

### Data and descriptive statistics

Table 1 presents some descriptive statistics about the clinical units under investigation and the whole DIEM, highlighting the characteristics of the hospitalized patients and the variables introduced as inputs in the model definition.

**Table 1:** Inputs adopted in the model specification

Variables	DIEM (%)	Internal medicine (%)	Cardiology (%)	Emergency medicine (%)	Geriatrics (%)	Respiratory diseases (%)	Neurology (%)	Oncology (%)
Age								
18–40 (years)	4.72	2.30	1.46	4.83	0.00	1.95	6.33	1.61
41–65 (years)	24.57	15.27	29.12	19.93	0.23	21.84	25.18	42.97
66–75 (years)	21.88	18.46	32.36	20.64	6.54	25.59	19.42	31.53
> 75 (years)	48.84	63.97	37.06	54.60	93.24	50.63	49.06	23.90
Presence of cancer	15.73	10.23	1.14	5.00	6.20	20.17	8.35	94.98
Sex (% male)	55.55	50.26	65.69	55.59	39.80	61.34	54.24	56.43
Transfers (every 100 hospitalizations)	–	0.09	0.2	0.16	0.04	0.06	0.09	0.08
Time slot								
Morning	33.20	16.50	74.10	12.70	19.50	31.30	34.20	57.20
Afternoon	48.80	60.60	16.70	68.40	56.80	54.80	43.70	33.50
Night	18.10	22.90	9.20	18.90	23.70	13.90	22.00	9.20
Day								
Monday	13.90	13.20	14.50	13.80	12.40	12.20	12.50	17.10
Tuesday	16.30	14.00	19.80	15.90	13.90	18.20	14.20	17.70
Wednesday	16.60	16.10	17.30	14.30	15.30	16.10	17.00	16.10
Thursday	15.80	14.20	20.20	15.50	15.90	15.30	16.40	16.70
Friday	16.30	15.90	17.80	15.10	15.60	17.50	14.20	16.70
Saturday	11.70	14.90	4.10	13.20	15.10	11.80	13.50	8.80
Sunday	10.15	11.70	6.30	12.20	11.80	8.80	12.10	7.00
Urgent hospitalization (direct)	12.8	2.40	4.00	3.40	1.80	21.30	12.40	18.50
Urgent hospitalization (from ER)	69.0	93.60	24.70	96.20	96.60	62.40	86.00	42.00
Planned hospitalization	18.2	4.10	71.40	0.40	1.60	16.30	1.60	39.60

The variable “transfers” is used exclusively in the micro-analysis (i.e., with the most representative medical units)

All the variables are expressed as average values, highlighting differences among our clinical units. Note that Table 1 considers exclusively the whole DIEM (first column), and then the medical units under investigations in the micro analysis, i.e., the most representative clinical units (internal medicine, cardiology, emergency medicine, geriatrics, respiratory diseases, neurology, and oncology). Tables 7 and 8 in Appendix show a more complete picture of our DIEM, showing all medical units.

Afterwards, considering the outcomes under investigation, Table 2 shows the percentage of hospitalizations in the clinical units, highlighting main differences. Considering the overall DIEM, 40% of the hospitalizations lasted more than the national average, and almost 3.74% were outliers. Note that, based on the aforementioned specification, these hospitalizations are a sub-sample of the whole set of longer hospitalizations. Finally, Table 2 illustrates other descriptive statistics on the DIEM units and their performance in 2018. In detail, the table shows the average LOS estimated on the basis of administrative data and the expected average LOS according to the national benchmarks. By comparing the two columns, we can identify the efficiency gap. For example, on average, the geriatrics unit has an overall LOS equal to 12.516 days for its hospitalizations, while the national average LOS was 9.808 days. This would translate into an average loss of more than 2 days for every hospitalization. Tables 9 and 10 in Appendix report all medical units, as well as the number of available beds and the number of hospitalizations in 2018. This information will be used to estimate the managerial impact of the proposed stratification rule. Lastly, Table 11 in Appendix show the correlation matrix among the main inputs introduced in the model definition.

**Table 2:** Clinical units and outcomes under investigation

Clinical unit	Hospitalization length^a^		Long hospitalizations (LOS > national average) (%)	Outlier hospitalizations (LOS > national threshold) (%)
	(Case study)	(National benchmark)		
Cardiology	5.352	5.764	19.38	1.62
Geriatrics	12.516	9.808	59.19	5.98
Internal medicine	10.076	9.744	41.66	4.23
Emergency medicine	9.387	9.412	39.14	4.38
Neurology	9.724	9.339	37.84	2.88
Oncology	11.533	10.717	36.75	2.41
Respiratory diseases	10.525	9.738	48.26	2.36
Total (DIEM)	9.891	9.219	40.06	3.74

^a^Average number of days

## Results

This section presents the results of our prediction models based on hospitalizations in 2018 (Table 3). In detail, taking the selected outcomes into account, the table displays the results in relation to the indexes defined in the previous section, as well as the number of observations (i.e., hospitalizations) used to validate every ANN (1/3 of the sample). Finally, in order to provide a more complete picture of our models and identify possible intervention strategies, Table 4 shows the weight of the covariates used in the ANNs, which is expressed as Garson indexes [35], and the expected sign of their contribution, which is expressed as a function of the NN synaptic weights [40].

**Table 3:** Results of our prediction models based on hospitalizations in 2018, considering the validation set (1/3 of the sample)

Outcome	Index	DIEM	Internal medicine	Cardiology	Emergency medicine	Geriatrics	Respiratory diseases	Neurology	Oncology
Long hospitalizations (> national average)	Incorrect classification	42.96%	47.66%	42.09%	42.09%	36.82%	41.67%	42.67%	39.16%
	Sensitivity	69.34%	68.98%	77.50%	63.51%	70.86%	62.07%	70.45%	67.21%
	Std. Err.	0.014	0.034	0.0.7	0.04	0.034	0.045	0.049	0.06
	[95% Conf. interval]	(0.666–0.72)	(0.618–0.755)	(0.668–0.861)	(0.552–0.713)	(0.635–0.775)	(0.523–0.709)	(0.598–0.797)	(0.54–0.787)
	Specificity	48.59%	40.46%	53.17%	54.22%	52.07%	54.84%	49.31%	57.14%
	Std. Err.	0.012	0.03	0.027	0.033	0.045	0.044	0.042	0.048
	[95% Conf. interval]	(0.462–0.51)	(0.345–0.467)	(0.476–0.506)	(0.475–0.609)	(0.428–0.612)	(0.456–0.638)	(0.409–0.578)	(0.471–0.667)
	False positive rate (type I error)	51.41%	59.54%	46.83%	45.78%	47.93%	45.16%	50.69%	42.86%
	False negative rate (type II error)	30.66%	31.02%	22.50%	36.49%	29.14%	37.93%	29.55%	32.79%
	Positive likelihood ratio	1.35	1.16	1.66	1.39	1.48	1.37	1.39	1.57
	Negative likelihood ratio	0.63	0.77	0.42	0.67	0.56	0.69	0.60	0.57
	Area under the curve	0.59	0.55	0.65	0.59	0.61	0.58	0.60	0.62
	Occurrence (outcome = 1)	1148	187	80	148	175	116	88	61
	Number of obs. (hospitalizations)	2819	449	411	373	296	240	232	166
Outlier hospitalizations (> national threshold)	Incorrect classification	47.85%	46.67%	18.49%	37.80%	40.54%	33.33%	44.40%	34.94%
	Sensitivity	62.73%	73.68%	71.43%	77.78%	77.78%	66.67%	85.71%	75.00%
	Std. Err.	0.046	0.101	0.171	0.098	0.098	0.192	0.132	0.216
	[95% Conf. interval]	(0.53–0.72)	(0.488–0.908)	(0.29–0.963)	(0.523–0.924)	(0.523–0.924)	(0.223–0.956)	(0.421–0.996)	(0.194–0.994)
	Specificity	51.72%	52.44%	81.68%	61.41%	58.27%	66.67%	54.67%	64.81%
	Std. Err.	0.01	0.024	0.019	0.026	0.029	0.031	0.033	0.037
	[95% Conf. interval]	(0.498–0.536)	(0.476–0.572)	(0.776–0.853)	(0.597–0.702)	(0.522–0.641)	(0.602–0.727)	(0.479–0.613)	(0.569–0.721)
	False positive rate (type I error)	48.28%	47.56%	18.32%	38.59%	41.73%	33.33%	45.33%	35.19%
	False negative rate (type II error)	37.27%	26.32%	28.57%	22.22%	22.22%	33.33%	14.29%	25.00%
	Positive likelihood ratio	1.30	1.55	3.90	2.02	1.86	2.00	1.89	2.13
	Negative likelihood ratio	0.72	0.50	0.35	0.36	0.38	0.50	0.26	0.39
	Area under the curve	0.57	0.63	0.77	0.70	0.68	0.67	0.70	0.70
	Occurrence (outcome = 1)	110	19	7	18	18	6	7	4
	Number of obs. (hospitalizations)	2,819	449	411	373	296	240	232	166

See “Empirical strategy” section for a clear explanation of these indexes

**Table 4:**
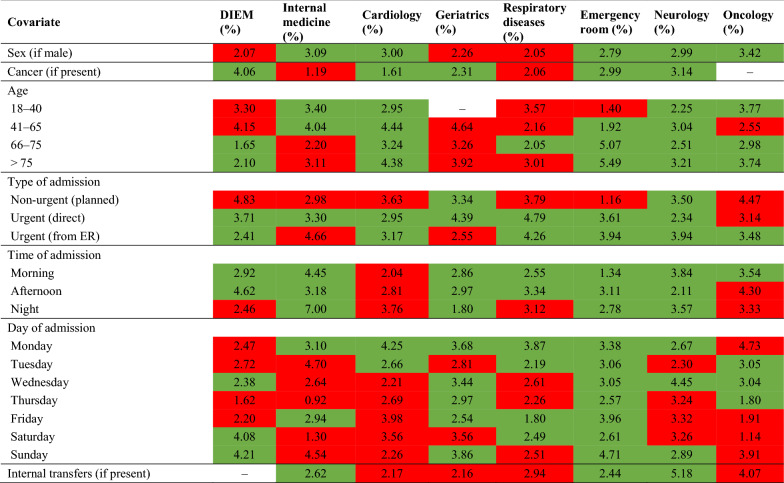
Garson indexes and covariates in the analysis with “long hospitalizations (LOS > national average)” as outcome

These indexes represent the contribution of each input introduced in the model definition to explain the outcomes under investigation (expressed as percentage weight)

Focusing on Table 3 and considering the macro analysis, the prediction ability of our model is equal to 69.34% (sensitivity) and 48.59% (specificity) in case of hospitalizations longer than the national average; while it is equal to 62.73% (sensitivity) and 51.72% (specificity) in case of hospitalizations longer than the national threshold. In other words, according to the results and the sample under investigation, our prediction models can identify correctly more than sixty percent of the hospitalizations with an expected excessive LOS. Focusing on the micro analysis, we observe a certain level of heterogeneity in our prediction models, which could be due to the internal organization of our medical units, and the impact of our inputs on the correct identification of the outcome under investigation (e.g., scheduling). Obviously, the values of sensitivity and specificity reflect the strategy adopted by authors in orienting the model [39]. Indeed, authors decided to maximize the ability of our prediction models in identifying correctly the subjects with hospitalizations longer than the selected values (i.e., national average or national threshold), guaranteeing the possibility for selected interventions aimed to save financial resources and, even more important, to improve the health conditions of these patients.

These interventions could be oriented to the internal organization of these hospitalizations, coherently with the insights proposed in Table 4. Indeed, depending on the selected outcome and the unit under investigation, the Table 4 indicates the contribution of every covariate in predicting that outcome (expressed as a percentage) and whether the impact is positive or negative (highlighted in the tables in green or red respectively). In other words, the red cells indicate that the variable can decrease the probability of obtaining the selected outcome, while the green cells indicate that the variable can increase the probability of obtaining the selected outcome. Note that we also consider the top 10 diagnoses as covariates (i.e., the diagnoses with the highest number of hospitalizations), although they are not presented here.

Focusing on patients’ admission to the DIEM (first column of Table 4), according to the estimated indexes, hospitalization during the night can contribute to the final outcome in the proportion of 2.46% (i.e., 2.46 is the weight of this covariate), reducing the likelihood of a LOS longer than the national average. Similarly, if the admission occurs during the afternoon, the contribution to the final outcome is almost double (i.e., 4.62%) and, even more importantly, its sign is opposite (i.e., increasing the probability of a LOS above the national average). In other words, at the DIEM level (i.e., macro analysis), the admission of patients in the morning or in the afternoon can increase the expected probability of a LOS longer than the national average, with the afternoon having the highest impact, while nighttime hospitalizations can decrease the likelihood of that outcome. Obviously, this information can be combined with the other information (e.g., main diagnosis, day and type of admission), so that managers and medical professionals may identify ways to reduce the current LOS, working both on current admission procedures and scheduling.

By looking at the covariates in the micro analysis (i.e., investigation of the single units), we can observe a certain degree of heterogeneity in our results, which is probably due to the different internal organization of the units and the clinical pathways adopted. Note that separate ANN frameworks have been run for each clinical unit; therefore, it is not surprising that the Garson indexes and relative contributions of the covariates are different among them, since they represent the detected heterogeneity. What about the managerial and clinical implications of our results?

### Clinical, managerial and economic implications of ANNs prediction models

Let us assume that the DIEM decides to adopt interventions necessary to improve its performance in 2019, based on the prediction models relying on the data extracted for 2018. In particular, let us imagine that the hospital management decides to use dedicated human resources to monitor all the patients identified as positive for the first outcome (i.e., hospitalizations with expected length above the national average and, thus, classified as longer) or, alternatively, to reorganize current hospital admissions according to the results displayed in Tables 4 (e.g., planning non-urgent hospital admissions on specific days). Finally, let us assume that the planned interventions are efficient, that is to say, they are successful in reducing the length of hospitalizations so as to reach the selected benchmark (i.e., the national average).

**Fig. 2:**
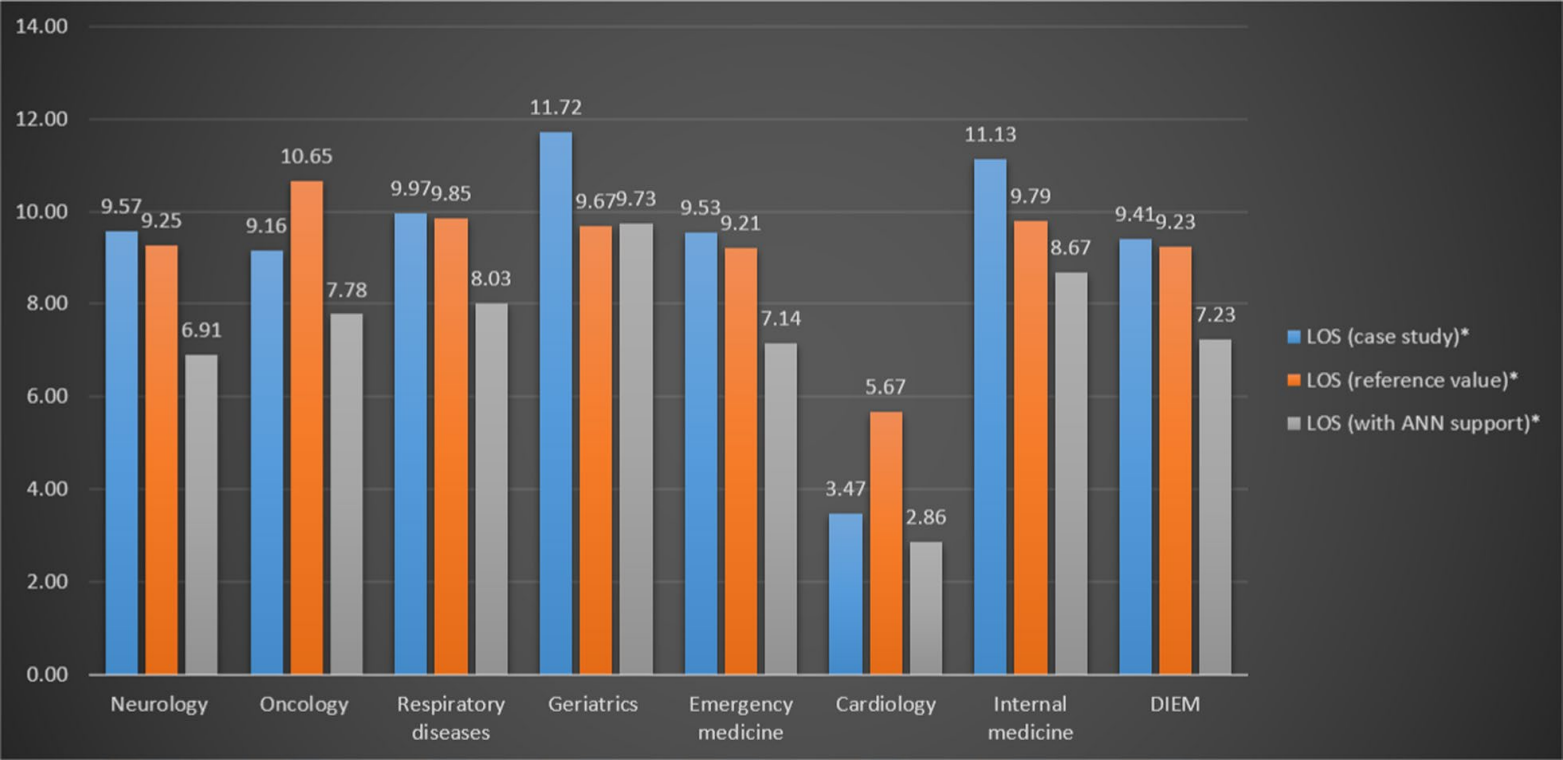
Simulation of the prediction models based on ANNs: LOS in 2019. *Average number of days

Figure 2 shows the results of this simulation following the aforementioned hypothesis (i.e., efficient interventions for all patients selected as positive to the first patient management outcome). In detail, the first column presents the observed average length of hospitalizations based on the 2019 activities, while the second column shows the expected average length according to the national benchmarks. Finally, the third column displays the theoretical LOS assuming that the DIEM carries out interventions on the patients identified as positive using the prediction models based on ANNs, and also assuming that the interventions are completely efficient (i.e., the DIEM reduces the length of all the selected hospitalizations so as to reach the national benchmark). By comparing the reported values, the readers can easily observe the impact of the supporting decision rule proposed here, which may significantly reduce the length of hospitalizations. On average, considering the whole department and the macro prediction model, LOS could decrease from 9.41 to 7.23 days, that is to say, on average, the department could make hospitalizations shorter by more than 2 days. Note that, by working on the patients identified as positive to the selected outcome and reducing their LOS, we may obtain a performance indicator that would be much better than the national average (i.e., 7.23 vs. 9.23).

The extra days gained thanks to this intervention could be used for other hospitalizations, lessening the current overcrowding and increasing the revenues of the department. Focusing on the latter outcome, Table 5 shows the economic impact of this improvement, reporting the number of additional hospitalizations and expected additional revenues. These revenues are estimated adopting the average hospitalization values collected in 2018, based on the specific DRG of these hospitalizations and current reimbursement values.

**Table 5:** Simulation of the prediction models: economic impact in 2019

Clinical unit	Additional hospitalizations	Additional revenues
Neurology	276	€ 1,062,940.31
Oncology	103	€ 455,507.30
Respiratory diseases	176	€ 687,060.68
Geriatrics	17	€ 58,250.64
Emergency medicine	361	€ 1,357,471.05
Cardiology	291	€ 1,615,504.42
Internal medicine	374	€ 1,227,035.69
Total (DIEM)	2650	€ 12,153,936.00

On average, adopting the prediction model presented here to identify patients at risk of prolonged LOS, the DIEM could expect additional revenues equal to more than 12 million Euro. Considering the single clinical units and using the prediction models showed in Table 5, we can observe that the most significant contribution is expected for Neurology, Emergency medicine, Cardiology and Internal medicine (i.e., economic impact higher than 1 million Euro). This is exactly the contribution of our prediction model based on ANNs to the management of patients: it can support medical professionals in the identification of the most critical cases and then, as they carry out the interventions that they deem necessary, it can improve outcomes and the efficiency of clinical units.

What about the second prediction model aimed at identifying outlier hospitalizations? Following the same approach as above and assuming effective interventions on all the patients with the identified outcome, our calculations reveal that the DIEM could shorten the average LOS up to 9.11 days, with a reduction equal to 0.30. This improvement could provide 288 additional hospitalizations and additional revenues amounting to € 1,318,428.27. Note that outlier hospitalizations only represent 3.74% of the total hospitalization sample under investigation (see Table 3), and this is why the expected impact is lower. Taking the clinical units into account, the most relevant economic implications are expected for Internal medicine, with 139 additional hospitalizations and expected additional revenues equal to € 456,320.00.

As highlighted in “Methods” section, the outliers are a sub-sample of the longer hospitalizations, and this is precisely why we decided to adopt both outcomes in our analysis, so that we would be able to estimate an interval for our potential interventions (i.e., between the benchmark and the threshold). Looking at the results, we can identify that interval, which is between € 1,318,428.27 and € 12,153,936.00 in terms of additional revenues and between 288 and 2650 in terms of additional days.

Finally, Table 6 focuses on the interventions that could be adopted to improve LOS, simulated by our prediction models for 2019 and considering three key diagnosis groups (i.e., heart failure, pneumonia, and sepsis). The second column of the table presents the observed average length of hospitalizations, while the third column displays the expected average length of hospitalizations according to the national benchmark. The fourth column, instead, shows the hypothetically achievable LOS if the DIEM adopts interventions on the patients identified by the prediction models based on ANNs, and assuming that these interventions are efficient (i.e., assuming that the DIEM can reduce the length of hospitalizations enough to reach the reference values).

**Table 6:** Simulation of the prediction models for 2019 according to main clinical diagnosis groups

Diagnosis	Number of hospitalizations	LOS (case study)^a^	LOS (national benchmark)^a^	LOS (with ANNs support)^a^	Additional days	Additional hospitalizations
Unspecified congestive heart failure (code 4280)	302	10.025	9.223	7.649	718	94
Left heart failure (code 4281)	223	9.494	8.658	6.879	583	85
Unspecified heart failure (code 4289)	27	9.775	9.321	7.250	68	9
Total (heart failure)	581	9.669	8.937	7.195	1437	200
Unspecified bacterial pneumonia (code 4829)	136	10.303	10.846	8.502	245	29
Unspecified bronchopneumonia (code 485)	151	10.952	10.801	8.470	375	44
Pneumonia, unspecified agent (code 486)	127	8.881	10.205	7.420	186	25
Total (pneumonia)	523	10.407	10.812	8.377	1062	127
Streptococcal (code 0380)	30	14.731	14.571	12.223	75	6
Unspecified staphylococcus (code 03810)	3	6.061	13.316	6.061	0	0
*Staphylococcus aureus* (code 03811)	41	22.161	14.798	14.163	328	23
Other staphylococci (code 03819)	19	20.266	14.432	13.326	132	10
Pneumococcal (code 0382)	9	19.385	14.323	15.122	38	3
Anaerobic (code 0383)	4	18.331	16.353	13.069	21	2
Unspecified gram-negative bacteria (code 03840)	21	15.551	13.316	10.903	98	9
Escherichia coli (code 03842)	148	12.566	13.542	10.819	258	24
Pseudomonas (code 03843)	26	13.586	14.297	12.407	31	2
Other gram-negative microorganisms (code 03849)	36	17.167	14.666	14.137	109	8
Other sepsis (code 0388)	19	14.751	13.956	11.783	56	5
Unspecified sepsis (code 0389)	119	13.609	13.699	11.636	235	20
Total (sepsis)	475	14.861	13.960	11.952	1382	116

^a^Average number of days

Focusing on these three key diagnosis groups rather than on the clinical units, Table 6 indicates where there might be opportunities to reduce LOS through the specific clinical pathways that regulate the treatment of patients. Obviously, this approach provides more easily readable and understandable outputs, shedding new light on the link between LOS and clinical pathways. For example, the prediction models are able to identify and support the appropriate monitoring of patients with heart failure and, assuming that the interventions are effective, 1437 additional days might become available for hospitalizations, which means 200 additional hospitalizations (considering the average LOS for this diagnosis group). According to our data, 90% of the additional days and hospitalizations are concentrated in two specific types of diagnosis: “unspecified congestive heart failure (code 4280)” and “left heart failure (code 4281)”. Therefore, medical professionals may work to modify the current clinical pathways for these two conditions, belonging to the macro classification “heart failure”, in order to optimize LOS.

## Discussion

The problem of correct inpatient scheduling is extremely significant for healthcare management [41, 42]. On the one hand, extended LOS duration can have negative effects on the supply of healthcare treatments, reducing patient accessibility. On the other hand, there can also be negative effects on the budget of healthcare suppliers, creating missed opportunities to increase hospital revenues by means of other treatments and/or additional hospitalizations. Finally, considering the market of healthcare services, social planners would have to tackle the consequences of lower supplier competitiveness, which could be even more relevant if we consider the current regulations on patients’ rights in cross-border healthcare (i.e., Directive 2011/24/EU of the European Parliament and of the Council of 9 March 2011).

According to our evidence, we can identify the main areas where managers could implement an internal re-organization to increase hospital’s efficiency and the effectiveness of its treatments, as well as the expected economic outcomes of these interventions. Indeed, the interpretation of the collected results can shed new light on the internal organization of medical units and its role as LOS driver. For instance, at macro level (i.e., considering the DIEM), results suggest that hospitalizations during the weekend (i.e., Saturday or Sunday) can have a negative impact on the expected LOS, increasing its probability of being longer than the national average. At the same time, *ceteris paribus*, hospitalizations during the night can decrease this probability. These insights could be clearly explained by the internal organization of these medical units and the impossibility to supply promptly treatments and interventions to hospitalized patients (e.g., during the weekend). At the same time, considering hospitalization during the night, we can imagine there could be more opportunities to plan properly patients’ therapeutic path, as well as more possibilities to pay appropriate attention to their diagnosis and the successive interventions. This could be explained by a lower demand of care in this specific moment (i.e., during the night), which can lead to minimize waste of time and resources in the initial steps.

Nevertheless, managers cannot control all LOS drivers. Results suggest that planned hospitalizations have a longer LOS, which is perfectly coherent with the type of access and it is outside the control of local management. With respect to this specific point, we might support the necessity to coordinate the territorial competence of ERs and the supply of treatments by policy makers, so that there could be an equilibrium between planned and urgent hospitalizations (according to Table 1, the number of non-urgent hospitalizations is less than 20%). Indeed, in order to contain the health expenditure, the number of medical centers and the supply of treatments have decreased significantly in the last years, creating a congestion effect that has increased the waiting lists and the access to the medical facilities by urgent cases (up to 80% in our case study).

These results are coherent with previous findings. On the one hand, literature emphasizes that medical units have to take care of urgent admissions and this adds a lot to the resulting inpatient pathway [43]. On the other hand, there are factors that strictly depend on the organizational process that could affect LOS [44, 45]. Indeed, coherently with our results, literature suggests that much of the variation in hospitals’ LOS is not attributable to patient illness, but rather it is due to differences in practice style [46] and it is potentially avoidable [47]. Moreover, as highlighted in our results, daily and timely kinetics of admissions are a known factor linked to the duration of hospital stay [44, 48].

### Limits

Even though our results are interesting and may effectively support physicians in their daily professional activities, our work also has some limits due to the available data and main assumptions (i.e., reference values and interventions).

First of all, the administrative data should be supported by more precise clinical information, if available. Indeed, gathering clinical and administrative information might help improve the current prediction models, reducing the number of false positives (i.e., lower cost in the patient monitoring phase, since there would be no risk of hospitalizations that are “too long”), as well as the number of false negatives (i.e., patients that are not monitored since there are wrong assumptions on their expected LOS).

Secondly, our results are solely based on the data collected in the specific department under investigation, with its specific internal organization, which clearly affects the final outcomes. Accordingly, our approach might prove effective only if adopted by health providers with similar characteristics and clinical pathways. Accordingly, there are clear limits in the external validity of our evidence, which are driven by the sample, the specific internal organization and the socio-economic environment under investigation.

Finally, we work under the assumption that the reference values are realistic and achievable, which we cannot take for granted. Taking the first outcome into account (i.e., longer LOS), the adopted reference values are based on the national average according to the DRG classification. This means that they are estimated considering all hospitalizations in Italy, classified under that specific DRG code upon discharge and assuming homogeneous procedures that could make the collected values comparable. Therefore, even if the data could be normalized to avoid outliers, the reference values might still be influenced by the sample selection, i.e., by the heterogeneity among regional healthcare systems and the specific procedures adopted by providers. If data become available, our DIEM should be compared with other departments that work within the same regional healthcare system and/or other comparable providers, so as to identify more realistic reference values.

### Future research

Although our results can shed new light on these complex systems, showing how they may support managers in dealing with LOS, there are limits to the present analysis and ample opportunities to further develop this research topic. Depending on data availability, it might be worthwhile to analyze the whole hospitalization path of patients, i.e., from admission to discharge, as well as the cases of readmission. In particular, the adopted clinical pathways might be investigated in greater depth, clustering observations according to patient diagnosis and controlling for transfers among clinical units and the measures used to coordinate such transfers.

## Conclusion

Adopting available national reference values and focusing on a Department of Internal and Emergency Medicine (DIEM) located in the North-West of Italy, this work assesses prediction models of hospitalizations with LOS longer than the selected benchmarks and thresholds. The prediction models investigated in this case study are based on Artificial Neural Networks (ANNs) and, according to our results, they might provide administrators and medical professionals with an effective tool to predict the outcome of hospital admissions and design interventions to improve hospital efficiency. Indeed, assuming effective interventions on all the subjects identified by the prediction models, the management of the DIEM could reduce the average LOS by up to 2 days, guaranteeing more than 2000 additional hospitalizations in a year. An innovative approach that might be based on smart solutions (e.g., a mobile app) to customize the stratification of patients admitted to the DIEM and to support the professionals’ decision making.

## Data Availability

The datasets generated and analysed during the current study are not publicly available but could be available on reasonable request.

## Appendix

See Tables 7, 8, 9, 10, 11.

**Table 7:** Clinical units and inputs (first part: patient characteristics and nature of hospitalization)

Clinical unit	Age (mean value)	Presence of cancer (%)	Sex (% male)	Transfers (mean value)^a^	Urgent hospitalization (direct) (%)	Urgent hospitalization (from ER) (%)	Planned hospitalization (%)
Cardiology	70	1.14	65.69	0.20	4.0	24.7	71.4
Hematology	58	91.26	50.00	0.04	45.1	15.0	39.9
Geriatrics	86	6.20	39.80	0.04	1.8	96.6	1.6
Infectious diseases	58	7.13	63.31	0.04	32.1	58.5	9.4
Internal medicine	77	10.23	50.26	0.09	2.4	93.6	4.1
Emergency medicine	73	5.00	55.59	0.16	3.4	96.2	0.4
Nephrology	67	7.46	49.75	0.11	28.9	63.2	8.0
Neurology	71	8.35	54.24	0.09	12.4	86.0	1.6
Coronary care	69	1.81	69.42	0.96	48.7	51.1	0.2
Gastroenterology	68	14.97	48.73	0.08	3.8	82.5	13.7
Oncology	67	94.98	56.43	0.08	18.5	42.0	39.6
Respiratory diseases	74	20.17	61.34	0.06	21.3	62.4	16.3
Rheumatology	63	0.00	18.18	0.00	18.2	31.8	50.0
Total (DIEM)	72	15.73	55.55	0.15	12.8	69.0	18.2

^a^Number of transfers every 100 hospitalizations

**Table 8:** Clinical units and inputs (second part: time and day of hospitalization)

Clinical unit	Time slot			Day						
	Morning (%)	Afternoon (%)	Night (%)	Monday (%)	Tuesday (%)	Wednesday (%)	Thursday (%)	Friday (%)	Saturday (%)	Sunday (%)
Cardiology	74.1	16.7	9.2	14.5	19.8	17.3	20.2	17.8	4.1	6.3
Hematology	47.2	48.6	4.2	14.3	16.8	17.1	17.5	19.9	9.4	4.9
Geriatrics	19.5	56.8	23.7	12.4	13.9	15.3	15.9	15.6	15.1	11.8
Infectious diseases	31.2	56.4	12.4	12.8	18.9	14.9	16.8	18.9	9.6	8.2
Internal medicine	16.5	60.6	22.9	13.2	14.0	16.1	14.2	15.9	14.9	11.7
Emergency medicine	12.7	68.4	18.9	13.8	15.9	14.3	15.5	15.1	13.2	12.2
Nephrology	29.9	57.2	12.9	16.4	14.4	17.4	11.9	17.9	13.4	8.5
Neurology	34.2	43.7	22.0	12.5	14.2	17.0	16.4	14.2	13.5	12.1
Coronary care	32.4	32.0	35.6	16.3	17.1	15.9	9.5	13.3	14.9	13.1
Gastroenterology	14.3	60.2	25.5	15.3	14.6	14.6	13.7	15.9	13.4	12.4
Oncology	57.2	33.5	9.2	17.1	17.7	16.1	16.7	16.7	8.8	7.0
Respiratory diseases	31.3	54.8	13.9	12.2	18.2	16.1	15.3	17.5	11.8	8.8
Rheumatology	9.1	86.4	4.5	22.7	9.1	22.7	18.2	18.2	0.0	9.1
Total (DIEM)	33.2	48.8	18.1	13.9	16.3	16.0	15.8	16.3	11.7	10.1

**Table 9:** Clinical units and outcomes under investigation

Clinical unit	Long hospitalizations (LOS > national average) (%)	Outlier hospitalizations (LOS > national threshold) (%)
Cardiology	19.38	1.62
Hematology	47.55	8.04
Geriatrics	59.19	5.98
Infectious diseases	39.83	3.56
Internal medicine	41.66	4.23
Emergency medicine	39.14	4.38
Nephrology	56.22	10.95
Neurology	37.84	2.88
Coronary care	42.05	3.22
Gastroenterology	33.76	0.96
Oncology	36.75	2.41
Respiratory diseases	48.26	2.36
Rheumatology	59.09	4.55
Total (DIEM)	40.06	3.74

**Table 10:** Clinical units and managerial information

Clinical unit	Hospitalization length (case study)^a^	Hospitalization length (national benchmark)^a^	Beds	Number of hospitalizations
Cardiology	5.352	5.764	28	1713
Hematology	18.699	16.039	15	300
Geriatrics	12.516	9.808	29	875
Infectious diseases	13.188	11.940	22	525
Internal medicine	10.076	9.744	37	1303
Emergency medicine	9.387	9.412	25	1090
Nephrology	14.364	9.333	8	218
Neurology	9.724	9.339	23	706
Coronary care	6.340	8.208	–	81
Gastroenterology	7.861	8.566	8	328
Oncology	11.533	10.717	18	561
Respiratory diseases	10.525	9.738	23	727
Rheumatology	13.997	9.387	2	30
Total (DIEM)	9.891	9.219	237	8457

^a^Average number of days

**Table 11:** Correlation matrix

Variables	(1)	(2)	(3)	(4)	(5)	(6)	(7)	(8)	(9)	(10)	(11)	(12)	(13)	(14)	(15)	(16)	(17)	(18)	(19)
Sex^(1)^	1																		
Afternoon^(2)^	− 0.034	1																	
	(0.002)																		
Night^(3)^	− 0.033	− 0.460	1																
	(0.002)	(0.000)																	
Morning^(4)^	0.063	− 0.683	− 0.335	1															
	(0.000)	(0.000)	(0.000)																
Monday^(5)^	0.010	− 0.004	− 0.001	0.005	1														
	(0.345)	(0.751)	(0.905)	(0.663)															
Tuesday^(6)^	0.009	− 0.008	− 0.059	0.057	− 0.178	1													
	(0.426)	(0.465)	(0.000)	(0.000)	(0.000)														
Wednesday^(7)^	− 0.003	− 0.003	− 0.034	0.031	− 0.176	− 0.193	1												
	(0.807)	(0.821)	(0.002)	(0.005)	(0.000)	(0.000)													
Thursday^(8)^	− 0.003	− 0.026	− 0.005	0.032	− 0.174	− 0.191	− 0.189	1											
	(0.770)	(0.016)	(0.650)	(0.004)	(0.000)	(0.000)	(0.000)												
Friday^(9)^	0.000	0.014	− 0.017	− 0.001	− 0.177	− 0.194	− 0.192	− 0.190	1										
	(0.995)	(0.205)	(0.123)	(0.939)	(0.000)	(0.000)	(0.000)	(0.000)											
Saturday^(10)^	− 0.012	0.018	0.084	− 0.088	− 0.146	− 0.160	− 0.158	− 0.157	− 0.160	1									
	(0.262)	(0.100)	(0.000)	(0.000)	(0.000)	(0.000)	(0.000)	(0.000)	(0.000)										
Sunday^(11)^	− 0.002	0.012	0.053	− 0.057	− 0.135	− 0.148	− 0.147	− 0.145	− 0.148	− 0.122	1								
	(0.831)	(0.257)	(0.000)	(0.000)	(0.000)	(0.000)	(0.000)	(0.000)	(0.000)	(0.000)									
Planned^(12)^	0.061	− 0.255	− 0.207	0.441	0.015	0.066	0.049	0.053	0.021	− 0.132	− 0.107	1							
	(0.000)	(0.000)	(0.000)	(0.000)	(0.183)	(0.000)	(0.000)	(0.000)	(0.057)	(0.000)	(0.000)								
Urgent—direct^(13)^	0.035	− 0.008	− 0.059	0.057	0.019	0.013	− 0.006	0.010	0.041	− 0.031	− 0.058	− 0.180	1						
	(0.001)	(0.467)	(0.000)	(0.000)	(0.087)	(0.233)	(0.601)	(0.379)	(0.000)	(0.004)	(0.000)	(0.000)							
Urgent—ER^(14)^	− 0.076	0.216	0.218	− 0.408	− 0.026	− 0.062	− 0.034	− 0.051	− 0.045	0.132	0.124	− 0.690	− 0.576	1					
	(0.000)	(0.000)	(0.000)	(0.000)	(0.016)	(0.000)	(0.002)	(0.000)	(0.000)	(0.000)	(0.000)	(0.000)	(0.000)						
Cancer^(15)^	0.017	− 0.009	− 0.077	0.072	0.029	0.015	− 0.001	0.010	0.008	− 0.036	− 0.036	0.144	0.132	− 0.213	1				
	(0.120)	(0.434)	(0.000)	(0.000)	(0.007)	(0.162)	(0.962)	(0.372)	(0.438)	(0.001)	(0.001)	(0.000)	(0.000)	(0.000)					
18–40 years^(16)^	− 0.008	0.011	0.001	− 0.012	− 0.001	0.008	− 0.009	0.002	0.014	− 0.015	− 0.001	− 0.039	0.076	− 0.022	− 0.011	1			
	(0.483)	(0.3109	(0.938)	(0.254)	(0.910)	(0.493)	(0.414)	(0.865)	(0.195)	(0.180)	(0.935)	(0.000)	(0.000)	(0.040)	(0.326)				
41–65 years^(17)^	0.096	− 0.044	− 0.033	0.074	0.002	0.021	− 0.012	− 0.008	0.010	− 0.002	− 0.014	0.089	0.135	− 0.171	0.110	− 0.127	1		
	(0.000)	(0.000)	(0.003)	(0.000)	(0.852)	(0.054)	(0.257)	(0.486)	(0.368)	(0.842)	(0.215)	(0.000)	(0.000)	(0.000)	(0.000)	(0.000)			
66–75 years^(18)^	0.090	− 0.029	− 0.030	0.055	0.009	0.003	0.019	0.005	0.007	− 0.022	− 0.028	0.116	0.025	− 0.115	0.067	− 0.118	− 0.302	1	
	(0.000)	(0.008)	(0.006)	(0.000)	(0.386)	(0.812)	(0.085)	(0.621)	(0.517)	(0.040)	(0.010)	(0.000)	(0.023)	(0.000)	(0.000)	(0.000)	(0.000)		
> 75 years^(19)^	− 0.154	0.057	0.053	− 0.104	− 0.009	− 0.023	− 0.001	0.001	− 0.020	0.027	0.035	− 0.155	− 0.169	0.252	− 0.146	− 0.217	− 0.558	− 0.517	1
	(0.000)	(0.000)	(0.000)	(0.000)	(0.407)	(0.032)	(0.918)	(0.905)	(0.063)	(0.015)	(0.001)	(0.000)	(0.000)	(0.000)	(0.000)	(0.000)	(0.000)	(0.000)	

Pairwise correlation coefficients with significance level in parentheses

## Abbreviations

LOS: Length of stay
ANNs: Artificial Neural Networks

## Footnotes

1 In this work, we have empirically found that the optimum number of hidden neurons is 5.

2 The tansigmoidal function has a logsigmoidal form with a codomain range from − 1 to + 1, while the logsigmoidal function’s codomain ranges from 0 to + 1.
